# Expression of OCT-4 and SOX-2 in Bone Marrow-Derived Human Mesenchymal Stem Cells during Osteogenic Differentiation

**DOI:** 10.3889/oamjms.2016.008

**Published:** 2016-01-18

**Authors:** Igor Matic, Maja Antunovic, Sime Brkic, Pavle Josipovic, Katarina Caput Mihalic, Ivan Karlak, Alan Ivkovic, Inga Marijanovic

**Affiliations:** 1 *Division of Biology, Department of Molecular Biology, Faculty of Science, University of Zagreb, Zagreb, Croatia*; 2 *Department of Traumatology, University Hospital Sestre Milosrdnice, Zagreb, Croatia*; 3 *Clinical Hospital Sveti Duh, Zagreb, Croatia*

**Keywords:** pluripotency markers, human mesenchymal stem cells, osteodifferentiation

## Abstract

**AIM:**

Determine the levels of expression of pluripotency genes OCT-4 and SOX-2 before and after osteogenic differentiation of human mesenchymal stem cells (hMSCs).

**METHODS:**

Human MSCs were derived from the bone marrow and differentiated into osteoblasts. The analyses were performed on days 0 and 14 of the cell culture. *In vitro* differentiation was evaluated due to bone markers – alkaline phosphatase (AP) activity and the messenger RNA (mRNA) expression of AP and bone sialoprotein (BSP). The OCT-4 and SOX-2 expression was evaluated at mRNA level by real-time qPCR and at protein level by immunocytochemistry.

**RESULTS:**

In vitro cultures on day 14 showed an increase in AP activity and upregulation of AP and BSP gene expression. OCT-4 and SOX-2 in undifferentiated hMSCs on day 0 is detectable and very low compared to tumor cell lines as a positive control. Immunocytochemistry detected OCT-4 in the cell nuclei prior (day 0) and post differentiation (day 14). On the same time points, cultures were negative for SOX-2 protein.

**CONCLUSION:**

Messenger RNA for pluripotency markers OCT-4 and SOX-2 isolated from hMSCs was less present, while OCT-4 protein was detected in cell nuclei prior and post differentiation into osteoblast lineage.

## Introduction

Molecular mechanisms regulating proliferation and multipotency of MSCs have not been well understood. In contrast, the molecular basis of proliferation and multipotency of embryonic stem cells (ESCs) is in the focus of research for a long time and it is described in detail. Expression of the three transcription factors, OCT-4, SOX-2 and NANOG, is essential for the major properties of stem cells, self-renewal and pluripotency [1, 2].

OCT-4 belongs to the family of Pou-domain transcriptional factors, and it is found in developing embryos, developing endoderm as well as developing neurectoderm [3–5]. OCT-4 together with SOX-2 up-regulates the expression of *NANOG* [6]. OCT-4 is a dose-depended pluripotency regulator that controls lineage commitment of ESCs [7]. *OCT-4* gene is expressed in tumor cells as well, but has not been found in differentiated tissues [8–10].

*OCT-4* expression has been confirmed in bone marrow (protein and mRNA level) [11], dental pulp [12], heart, liver [13] and adipose tissue-derived stem cells [14].

SOX-2 is a member of the SRY-related HMG-box (SOX) transcription factor family with a diverse role in stem cell potency and maintenance, embryonic development and cancer [15–18]. It is closely co-regulated alongside core pluripotency factors OCT-4 and NANOG in ESCs, embryonic carcinoma cells (ECCs) and induced pluripotent stem cells (iPSCs) [18–20]. Recently, SOX-2 has been implicated in the maintenance and differentiation of adult stem cells. *SOX-2* expression has been reported in bone marrow, neuronal tissues and sensory epithelia [11, 21].

It has been speculated that the same set of transcription factors plays an important role in the maintenance of multipotency and self-renewal of adult stem cells. Adult stem cells are present within the tissues with the purpose to repopulate them after injury or physiological loss. Human MSCs derived from bone marrow are progenitors that can differentiate into cells of multilineage, including osteogenic, chondrogenic, adipogenic and myogenic lineages [22–24]. Human MSCs, like human ESCs, are depended on fibroblast growth factor (FGF) to maintain self-renewal and pluripotency [25]. FGF inhibits differentiation by bone morphogenetic protein (BMP) signaling inhibition and sustain expression of OCT-4, SOX-2 and NANOG pluripotency associated genes [26]. Upon differentiation expression of pluripotency transcriptional factors should be downregulated [27]. However, there is a controversy among different research groups regarding the expression of pluripotency regulators in adult tissues [28]. Human MSCs exhibit heterogeneous characteristics with regard to morphology, proliferation rate and secreted factors as a consequence of variable gene expression pattern. Difference in gene expression is dependent of their intrinsic heterogeneity and culture conditions [29]. In particular, *ex vivo* expansion of hMSCs is used for cell therapy and tissue engineering. Therefore it is an important to characterize cells cultured *in vitro* for the expression of genes responsible for self-renewal ability in order to predict better their behavior.

In this study, we evaluated the expression of *OCT-4* and *SOX-2*, in cultured bone marrow-derived mesenchymal stem cells of human origin. There are no universal stem cell markers that are expressed in all types of stem cells [30]. For that reason it is crucial to determine specific stem cell markers for cells that can be used for clinical research, such are hMSCs. By determining the stem cells markers expression we would be able to estimate the quality of stem cells that must be associated with cell therapy efficacy. Therefore, we have examined changes of stem cell markers expression during osteodifferentiation of hMSCs. Here we report effects of osteodifferentiation on OCT-4 and SOX-2 mRNA and protein expression.

## Materials and Methods

Appropriate regulatory approval from Medical Ethics Committee of Clinic for Traumatology Zagreb was taken before initiating the experiments.

### Human mesenchymal stem cells, cell lines and culture conditions

The hMSCs were isolated and propagated using previously described methods (Romanov et al. 2005). Two human cancer cell lines were used in the experiment: glioma cell line A1235 and liver cell cancer line HepG2. The cells were kindly provided by Prof. M. Matulić, University of Zagreb, The Faculty of Science (Croatia). Both cell lines were cultured in a medium which contained Dulbecco’s Modification of Eagle’s Medium (Sigma-Aldrich) supplemented with 10% FBS (Gibco) and penicillin (100 U/ml)/streptomycin (100 µg/ml) (Sigma-Aldrich) at 37°C in a humidified atmosphere containing 5% CO_2_.

### In vitro differentiation of hMSCs into osteogenic lineage

Three or four passages of BMSCs were adjusted to a concentration of 47 500 cells/well in a 6-well plate (Corning) in proliferation medium DMEM supplemented with 10% FBS, penicillin/streptomycin (P/S) and 10 ng/ml FGF2. The cells were cultured in a humidified incubator (37°C, 5% CO_2_) with renewal of the culture medium every 3 days. The cells were tested for their known ability to differentiate into osteogenic lineages by growing them in osteogenic induction medium when the plates were 100% confluent. Culture media was then replaced with osteogenic induction medium (αMEM 10% FBS + P/S + 50 μg/ml ascorbic acid + 4 mM β-glycerophosphate + 1 μM dexamethasone) after 24 hours and cells were maintained 14 more days under differentiating conditions. All the osteogenic induction factors were purchased from Sigma-Aldrich. Medium was replaced every 2 days. Process of differentiation was held for 14 days, and day 0 was marked as the day when differentiation medium was added.

### Determination and quantification of alkaline phosphatase (ALP) activity

After osteogenic differentiation, cells were washed in PBS, briefly fixed with citrate-acetone-formaldehyde fixative and ALP expression was assessed using the ALP staining kit (86R, Sigma-Aldrich) according to the manufacturer’s instructions. The results were recorded using a scanner. The alkaline phosphatase activity was assessed 14 days after osteogenic induction. Cell layers were twice washed in PBS and the activity was measured by using p-nitrophenyl phosphate (Sigma-Aldrich) as a substrate. Briefly, cells were incubated with p-nitrophenyl phosphate in substrate buffer (50 mM glycin, 1mM MgCl_2_, pH 10.5) at room temperature for 10 min. Supernatant was mixed with 1 ml 1M NaOH and absorbance at 405 nm was recorded [32]. Each experiment was done in triplicates and repeated at least three times.

### RNA extraction and real-time quantitative PCR

Total RNA was collected from 14 days old osteoblast cultures as well as from undifferentiated hMSCs and two human cancer cell lines using TRIzol reagent (Invitrogen Life Technologies) according to manufacturer’s instructions. Briefly, cell layers were washed with PBS and scrapped and homogenized in 1 ml TRIzol. RNA was treated with DNase I (Invitrogen) to remove genomic DNA and 3 µg of total RNA was reverse transcribed to cDNA using Superscript III First-Strand Synthesis System for RT-PCR (Invitrogen) according to the manufacturer’s instructions. Reverse transcription was performed in thermomixer (Eppendorf) at following conditions: 10 minutes at 20°C, 1 hour at 42°C, 5 minutes at 99°C and 5 minutes at 5°C. Gene expression levels were determined by real time-qPCR using Power SYBR Green Mastermix (Applied Biosystems). Primers (Sigma-Aldrich) used to determine gene expression levels were presented in Table 1. The PCR reaction conditions were as follows: 10 minutes at 95°C for 1 cycle, 15 seconds at 95°C and 1 minute at 60°C for 40 cycles. Quantitative RT-PCR was performed on 7500 Fast PCR system (Applied Biosystems). Expression levels were normalized to *β-actin*. Relative expression of target genes was calculated using the ΔΔCt method. Stem cell markers expression in differentiated cultures (day 14) was normalized to 1 as well as *BSP* and *AP* expression in 14 days old hMSCs cultures (day 14).

**Table 1:** Primers used for quantitative real-time PCR

Primers	forward	reverse
human P0U5F1	5’-GATCACCCTGGGTATACAC-3’	5’-GCTTTGCATATCTCCTGAAG-3’
human S0X2	5’-ATAATAACAATCATCATCGGCGG-3’	5’-AAAAAGAGAGAGGCAAACTG-3’
human IBSP	5’-GGAGACTTCAAATGAAGGAG-3’	5’-CAGAAAGTGTGGTATTCTCAG-3’
human ALPL	5’-TCTTCACATTTGGTGGATAC-3’	5’-ATGGAGACATTCTCTCGTTC-3’
human ACTB	5’-GACGACATGGAGAAAATCTG-3’	5’-ATGATCTGGGTCATCTTCTC-3’

### Immunocytochemistry

Cells were seeded in 35 mm Petri dishes. Medium was removed, cell layers were rinsed in ice cold PBS, fixed in 4% paraformaldehyde for 15 min and washed in PBS. Cells were permeabilized with 0.25% Triton-X-100 (Packard) and blocked with 1% bovine serum albumin (BSA) in PBS for 30 min. Used primary antibodies were ab19857 rabbit polyclonal anti-Oct4 (Abcam) diluted 1:100, ab97959 rabbit polyclonal anti-Sox2 (Abcam) diluted 1:100 in 1% BSA and ab21624 rabbit polyclonal anti-Nanog (Abcam) diluted 1:100. All antibodies were applied for 1 hour at room temperature. Secondary antibody (Alexa Fluor® 488 Donkey Anti-Rabbit IgG (H+L), Invitrogen) were applied for 1 hour at room temperature in a dark room. Cells were further counterstained for 1 min with Hoechst solution (1µg/ml) and imaged with CCD camera on Olympus BX51.

### Statistical analysis

A statistical analysis of the data was performed using a one-way ANOVA analysis of variance followed by a Duncan test using the *STATISTICA 12* program (StatSoft, Inc.,). Statistical significance was set at *p* < 0.05.

## Results

**Figure 1:**
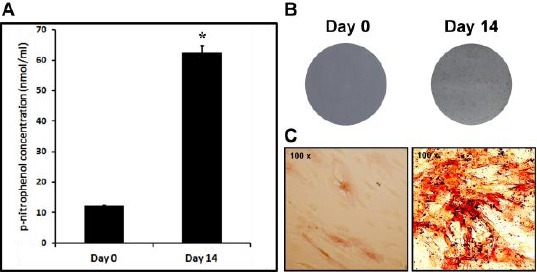
High alkaline phosphatase activity is an indication of successful differentiation of hMSCs into osteoblast after 14 days of osteoinduction. The alkaline phosphatase (AP) activity was measured by using p-nitrophenyl phosphate as a substrate. This quantitative method has shown higher alkaline phosphatase activity in differentiated hMSC cultures (Day 14) than in undifferentiated cultures (Day 0) (A). This result has been confirmed by AP cytochemical staining (B, C). Red dye deposits indicate sites of alkaline phosphatase activity in hMSC cultures on day 14 and just a few undifferentiated hMSCs were red. The data are expressed as the means + SD of triplicate determination (*P<0.05).

Bone marrow-derived human mesenchymal stem cells were propagated *in vitro* and osteogenic differentiation was induced. The level of differentiation was assessed by analysis of bone markers. Early marker of osteoblast differentiation is the activity of alkaline phosphatase (AP). The AP activity was evaluated before and after osteogenic differentiation by cytochemical staining. The quantitative method where p-nitrophenil phosphate was used as a substrate, showed 6-fold increase in AP activity on day 14 compared to undifferentiated culture (Figure 1A).

**Figure 2:**
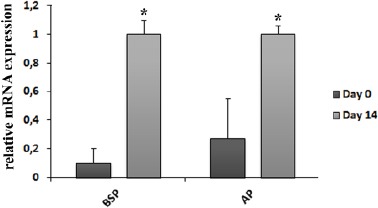
Relative mRNA expressions of osteogenic markers detected by real-time quantitative PCR on day 0 and day 14. The relative gene expression of BSP and AP was analyzed by ΔΔ cycle threshold method and the values were normalized to β-actin expression. Those values were then normalized to hMSCs after 14 day of osteoinduction. Data are represented as an average of three independent patient samples and error bars represent mean value + SD (*P<0.05). Abbreviations: BSP, bone sialoprotein; AP, alkaline phosphatase; hMSCs, human Mesenchymal Stem Cells.

These findings are in coherence with results obtained from the cytochemical staining of day 0 and day 14 cultures (1B, 1C). Further analysis of differentiation included quantitative analysis of mRNA levels by real-time qPCR. We analyzed expression of two genes, alkaline phosphatase (AP) as an early marker of osteoblast differentiation and bone sialoprotein (BSP) as an intermediate to late marker of osteoblast differentiation with the peak of expression on day 14. The 5-fold increase of AP mRNA and 10-fold increase of BSP mRNA confirmed that MSCs differentiated into osteoblast lineage after 14 days of osteogenic induction (Figure 2).

To estimate the expression of pluripotency genes, we analyzed *OCT-4* and *SOX-2* mRNA levels by real-time qPCR prior and post differentiation of human MSCs. To confirm data on protein level, we performed immunostaining for OCT-4 and SOX-2. Data were compared with positive controls, tumor cell lines positive for OCT4/SOX2 expression, glioma cell line A1235 and liver cell cancer line HepG2.

**Figure 3:**
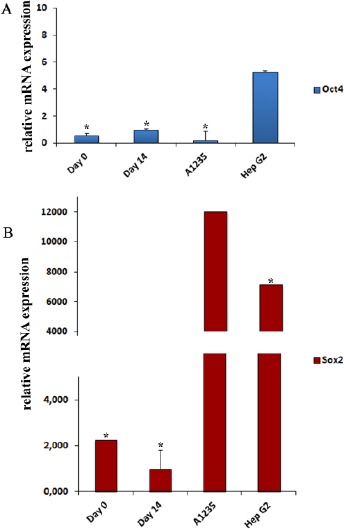
Expression levels of OCT-4 and SOX-2 genes in hMSCs after 14 days of osteoinduction, undifferentiated hMSCs and two human cancer cell lines A1235 and HepG2 detected by real-time qPCR. OCT-4 is expressed in undifferentiated hMSC (Day 0) but expression of OC-T4 gene does not go down after osteoinduction on day 14. Relative mRNA expression of OCT-4 in Hep G2 cell line was 9.9 x more expressed than in undifferentiated hMSCs (A). Transcription factor SOX-2 is expressed in undifferentiated hMSCs and its expression goes down after osteoinduction on day 14. Relative mRNA expression of SOX-2 in Hep G2 cell line was 5321.9 x more expressed than in undifferentiated hMSCs while in glioblastoma A1235 cell line was 3169.8 x respectively (B). The quantity of gene expression was normalized to β-actin to determine the quantitative differences. Data are represented as an average of three independent patient samples and error bars represent mean value + SD. In the columns marked with asterisk * the mean values are significantly different then control (Hep G2) (A) or A1235 and Hep G2 (B), respectively, according to Duncan test (*P<0.05).

**Figure 4:**
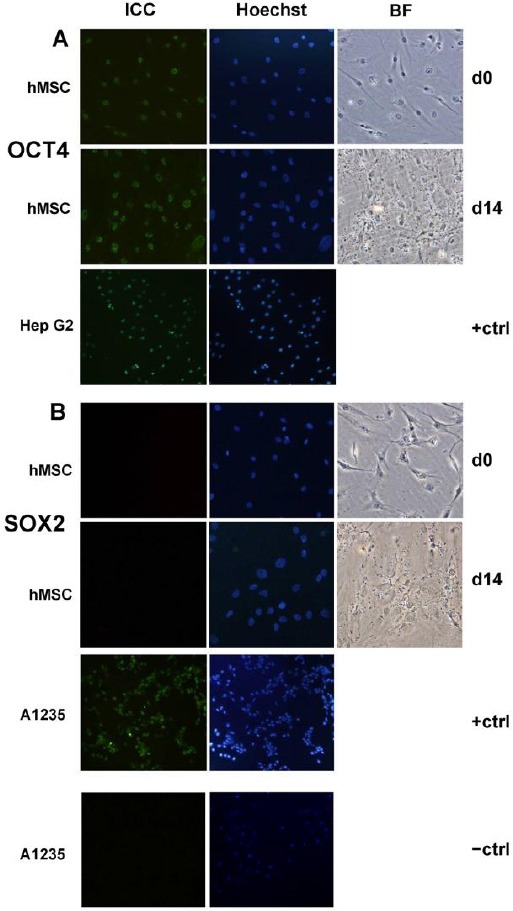
*Immunostaining for OCT-4 did not show any changes in OCT-4 protein level in osteo-differentiated hMSC cultures compared to undifferentiated. Osteoblast and undifferentiated hMSCs were both positive for OCT-4 expression and had a nuclear localization of OCT-4 (A). Immunofluorescence staining for SOX-2 did not confirm levels of SOX-2 protein in osteo-differentiated hMSCs (Day 14) nor in undifferentiated hMSCs (Day 0) (B). Liver cell cancer line HepG2 has been used as positive control for OCT-4 expression. Glioblastoma A1235 cell line has been used as positive control for SOX-2 expression and for no primary antibody control (-ctrl) as well. Nuclei were stained with Hoechst (200x, CCD camera on Olympus BX51). Abbreviations: ICC, immunocytochemistry; BF, bright field; d0, the day when differentiation medium was added; d14, 14th day of osteogenic differentiation*.

**Figure 5:**
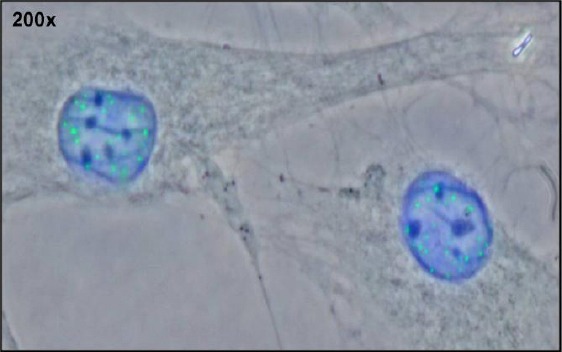
*Fluorescence microscopy image of undifferentiated hMSCs immunostained for OCT-4. Immunofluorescence staining for Hoechst (blue) and OCT-4 (green) indicates a nuclear localization of OCT-4 protein (20x, CCD camera on Olympus BX51)*.

Expression of *OCT-4* mRNA was detected prior the differentiation and it unexpectedly increased 2-fold after differentiation. However, the levels of expression are very low when compared to OCT-4 positive tumor cell line HepG2 (Figure 3A). To confirm our findings on protein level we performed immunostaining and protein OCT-4 was detected in cultures before and after differentiation (Figure 4A). OCT-4 signal was localized in the cell nuclei as expected for transcription factor (Figure 5).

Expression of *SOX-2* mRNA was also detected in undifferentiated MSCs and after differentiation at day 14 was downregulated. Overall expression of *SOX-2* was extremely low when compared to positive control cell lines A1235 and HepG2. At protein level, SOX-2 was undetectable before and after differentiation. Therefore, findings at protein level did not reflect findings on mRNA level.

## Discussion

Human mesenchymal stem cells (hMSCs) provide an excellent source of multipotent progenitor cells that are present in bone marrow as well as in most of connective tissues. Due to their proliferation and differentiation capacity, they are able to differentiate toward multiple mesodermal lineages [13]. In this work, we have examined hMSCs osteogenic potential. Human MSCs were differentiated into osteoblasts by standardized method where hMSCs are treated with osteogenic inductors, ascorbic acid, β-glycerophosphate and dexamethasone [33]. Here, we have investigated and compared the change of osteogenic markers expression and the alteration of stem cell markers expression OCT-4 and SOX-2 during osteogenic differentiation of hMSCs.

We have followed the change of early and late osteogenic markers. Alkaline phosphatase was used as an early osteogenic marker and bone sialoprotein as late marker. Our results showed that on day 14 all observed osteogenic markers are highly expressed and hMSCs were differentiating towards osteoblast lineage. As previously described [34] we have also observed a few distinct alkaline phosphatase positive cells on day 0. In this report, we have investigated weather two stem cell markers, OCT-4 and SOX-2, known to regulate potency and self-renewal of embryonic stem cells, were expressed in hMSCs and how their expression changed after osteoinduction. OCT-4 and SOX-2 mRNA expression was examined at mRNA level by qPCR and at protein level by immunocytochemistry. Real-time qPCR revealed that OCT-4 and SOX-2 mRNAs are expressed in hMSCs and their expression is very low when compared with tumor cell lines used as a positive control. After differentiation the level of OCT-4 increased and the level of SOX-2 decreased. Immunocytochemical staining revealed that OCT4 protein is expressed in all hMSCs and stays expressed on day 14, localized in the nuclei. In contrast to previous observations [11], SOX2 have not been detected neither in hMSCs on day 0 nor in osteoblasts on day 14.

We demonstrate presence of stem cell markers OCT-4 and SOX-2 in human mesenchymal stem cells at mRNA level. Immunocytochemical analysis revealed OCT4 expression in nuclei but not in cytoplasm as previously has been demonstrated [34]. In past decade, many controversial results have been obtained regarding the expression and the role of stem cell transcriptional factors in adult stem cells. OCT-4 and SOX-2 were undoubtedly confirmed in some types of adult progenitor and multipotent stem cells [34–37]. They are expressed in tumor cells [38–40] and might be responsible for cancer stem cells resistance to chemotherapy [41]. Previous reports showed inconsistent results in the expression of stem cell markers in adult stem cells. Beltrami et al., as well as some other groups have confirmed OCT-4 and SOX-2 and NANOG stem cell markers expression in mesenchymal stem cells [11, 13, 34] but Pierantozzi et al. have not detected OCT-4 and NANOG in adult hMSCs [24]. Little is known about the role of OCT-4 and SOX-2 in adult stem cells. *OCT-4* gene knockdown promotes differentiation thereby that transcriptional factor play an important role in stem cell self-renewal [42]. We find that hMSCs express low levels of OCT-4, what is crucial for multipotency and self-renewal of these adult stem cells. It has been previously reported that increased expression of *OCT-4* causes increased differentiation efficiency towards osteoblasts [43].

Immunohistochemical staining did not confirm OCT4 protein *in vivo*, in bone marrow sections. There is a possibility that *ex vivo* expansion of human mesenchymal stem cells causes activation of endogenous *OCT-4* gene expression. Therefore, OCT-4 might play an important role for maintaining potency in somatic stem cells [28] including hMSC. Synthesis and degradation of OCT-4 protein is very rapid process and alteration of *OCT-4* transactivation potential in response to extracellular signals is reversible [44]. This kinetic is not completely understood yet, but might explain why very high OCT-4 expression is present in osteoblasts on day 14. Relative amounts of OCT-4 protein are essential for proper differentiation and early development of an organism because it determines developmental directions. Post-translational modification of OCT-4 by sumoylation can enhance OCT-4 stability, DNA binding and transactivation [45]. OCT-4 is ubiquitinated by Wwp2 and degradated during differentiation of embryonal carcinoma cell line but does not affect OCT-4 protein level in embryonic stem cells [46]. These post-translational modifications are important for OCT-4 stability, activity and degradation, so these mechanisms may maintain its quantity during differentiation of hMSCs.

SOX2 is a transcription factor co-expressed with OCT-4. It has been identified a crucial player in the maintenance and differentiation of adult stem cells such as in neural stem cells. Seo et al. showed that SOX-2 maintains self-renewal and proliferation of the osteoblast precursors [47]. SOX-2 inactivation in cultured primary osteoblasts has been shown to cause exhaustion of their proliferative ability and senescence [48]. Our findings support the existence of progenitors that express SOX-2 among the population of hMSCs, however the levels detected by qPCR may be under the detection limit of immunocytochemistry or posttransciptional regulation may be responsible for absence of protein. Downregulation of SOX-2 after the differentiation is consistent with suggested role of SOX-2.

Despite the fact that probably there is no universal stem-cell marker which could be applied to all types of stem cells [30], it is important to define stem cell markers specific for clinically relevant cell types such as MSCs. These markers could be used for the evaluation of stem cell differentiation potential. In this study, since hMSCs have well defined osteogenic markers, we have tried to define some stem cell markers in these cells. We have evaluated two potential candidates, OCT-4 and SOX-2. It is crucial to further examine their function in hMSCs as well as in osteoblasts. It is important to be able to evaluate the quality of stem cells, because the stem cell potential is certainly associated with the potential results of cell therapy.

One of the biggest problems in cell therapy is a potential malignant transformation of stem cells. It is well known that the stem and tumor cells in some aspects of their biology are very similar. During the process of the tumor stem cell dedifferentiation, re-expression of some stem cell markers occurs [40] and very often OCT-4 levels are dramatically increased in cancer cells [49]. Potentially, by monitoring the expression of OCT-4 gene in stem cells we could predict the behavior of cells in terms of malignant transformation. This is another reason why it is necessary to investigate the role of pluripotency genes in somatic stem cells.

In conclusion, bone marrow-derived mesenchymal stem cells that are expanded in culture in the presence of FGF2 clearly express low levels pluripotency markers OCT-4 and SOX-2 mRNA. Protein OCT-4 is detected in cell nuclei before and after differentiation while SOX-2 is not detected. Expression of OCT-4 is not desirable property of cells that are going to be applied in the clinical setting. We conclude that culture conditions do have an effect on stem cell marker expression and it is important to standardize cultivation conditions in order to better predict cell behavior. It is necessary to clarify better the role and mechanism of action of core pluripotency genes in mesenchymal stem cells following their cultivation *in vitro*.
